# WSRD-Net: A Convolutional Neural Network-Based Arbitrary-Oriented Wheat Stripe Rust Detection Method

**DOI:** 10.3389/fpls.2022.876069

**Published:** 2022-05-24

**Authors:** Haiyun Liu, Lin Jiao, Rujing Wang, Chengjun Xie, Jianming Du, Hongbo Chen, Rui Li

**Affiliations:** ^1^Institute of Intelligent Machines, Hefei Institutes of Physical Science, Chinese Academy of Science, Hefei, China; ^2^Science Island Branch, University of Science and Technology of China, Hefei, China; ^3^School of Internet, Anhui University, Hefei, China; ^4^Institutes of Physical Science and Information Technology, Anhui University, Hefei, China

**Keywords:** arbitrary-oriented, convolutional neural network, deep learning, wheat strip rust, detection

## Abstract

Wheat stripe rusts are responsible for the major reduction in production and economic losses in the wheat industry. Thus, accurate detection of wheat stripe rust is critical to improving wheat quality and the agricultural economy. At present, the results of existing wheat stripe rust detection methods based on convolutional neural network (CNN) are not satisfactory due to the arbitrary orientation of wheat stripe rust, with a large aspect ratio. To address these problems, a WSRD-Net method based on CNN for detecting wheat stripe rust is developed in this study. The model is a refined single-stage rotation detector based on the RetinaNet, by adding the feature refinement module (FRM) into the rotation RetinaNet network to solve the problem of feature misalignment of wheat stripe rust with a large aspect ratio. Furthermore, we have built an oriented annotation dataset of in-field wheat stripe rust images, called the wheat stripe rust dataset 2021 (WSRD2021). The performance of WSRD-Net is compared to that of the state-of-the-art oriented object detection models, and results show that WSRD-Net can obtain 60.8% AP and 73.8% Recall on the wheat stripe rust dataset, higher than the other four oriented object detection models. Furthermore, through the comparison with horizontal object detection models, it is found that WSRD-Net outperforms horizontal object detection models on localization for corresponding disease areas.

## Introduction

As a widely cultivated crop in the world, wheat is crucial for ensuring food security. Unfortunately, its production is limited by many diseases (Savary et al., 2012). Among these diseases, stripe rust is one of the main diseases of wheat (Chen et al., 2007; Wan et al., 2007), which is caused by the fungus *Puccinia striiformis* var. tritici (Moshou et al., 2004). Stripe rust occurs primarily on leaves, in which bright yellow pustules emerge at the early stage of the wheat, and the pustules are arranged as stripes in linear rows. Stripe rust damages wheat leaves and causes yield losses under severe epidemics. Therefore, early detection of wheat stripe rust is essential for sustainable agriculture. The traditional method of disease detection involves manual examination by either farmers or experts (Duveiller et al., 2007), which can be time-consuming and high labor-costing, proving infeasible for millions of small and medium-sized farms around the world. To identify the diseases based on early symptoms, various spectroscopic and imaging techniques have been proposed by many researchers (Huang et al., 2007; Belasque et al., 2008; Qin et al., 2009; Mahlein et al., 2013; Zhang et al., 2014; Barbedo et al., 2015). Although these techniques can make a relatively rapid diagnosis for crop diseases, expensive and bulky sensors are required for such method.

With the development of computer vision, different researchers have addressed identification of crop disease by computer vision technologies (Rastogi et al., 2015; Ma et al., 2017; Hossain et al., 2018; Jian-Jun et al., 2019; Larijani et al., 2019; Pandey et al., 2021). These methods use the images captured by the common cameras to identify crop diseases, thus getting rid of the constraints of time-cost and expensive and bulky sensors (Martinelli et al., 2015). However, these methods need to design manual feature extractors, and are only suitable for crop images in an ideal experimental environment.

In recent years, as a new breakthrough in the field of computer vision, deep learning methods have been used to deal with various problems in the agricultural field. Many researchers have utilized various deep learning techniques for identification of crop disease (Liang et al., 2019; Zeng and Li, 2020; Zhao et al., 2020). Amanda et al. (2017) applied transfer learning to train a deep convolutional neural network to identify cassava disease. Ferentinos (2018) trained and evaluated specific CNN architectures to form an automated plant disease detection and diagnosis system based on simple leave images of healthy and diseased crops. Joshi et al. (2020) proposed a convolutional neural network VirLeafNet to classify the leaves of *Vigna mungo* into healthy, mild disease, and severe disease categories. They segmented images to better extract features, and then augmented them to increase the number of images in dataset. Hence, robustness of the training model is enhanced.

**Figure 1:**
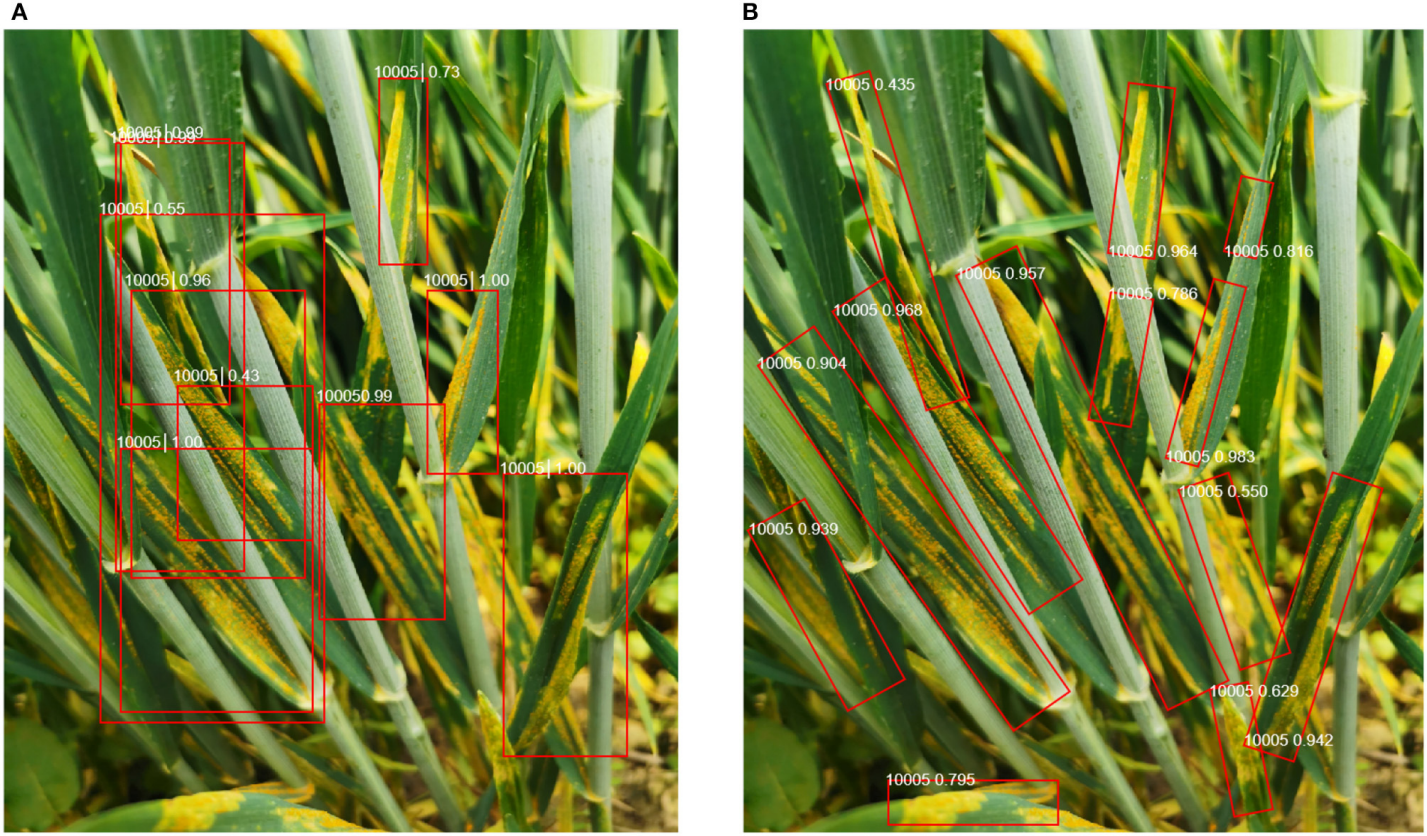
Comparison of horizontal object detection and oriented object detection results. **(A)** Horizontal object detection and **(B)** oriented object detection.

However, the abovementioned works just realized the identification of diseases but paid no attention to where the diseases are. The detection of disease region in field scenarios is also very important. It can help farmers and agricultural experts better identify the specific location of crop diseases in complex field scenarios (especially those disease symptoms that are not obvious and easy to be confused in the image). In addition, a complex field background can make it challenging to recognize the crop disease and misguide the classifier when the target disease region is not salient. Therefore, crop disease detection can also improve classification performance by removing irrelevant background features. Of course, many crop disease detection researches have been proposed (Inkyu et al., 2016). (Fuentes and Park, 2017) used the state-of-the-art generic object detection approaches, namely, Faster RCNN and SSD, for localizing crop leaves and disease spots with good performance. Lu et al. (2017) presented an in-field automatic wheat disease diagnosis system based on a weak supervised deep learning framework, which integrates wheat disease identification and localization for disease areas with only image-level annotation for training images in wild conditions. Zhang et al. (2021) designed a multi-feature fusion Faster R-CNN method (MF^3^ R-CNN) to detect soybean leaf disease in complex scenes. They also developed a synthetic soybean leaf disease image dataset to tackle the problem of insufficient datasets. Although these methods can perform well on crop disease detection tasks, it is still challenging to obtain good performance from wheat stripe rust images in wild scenarios containing various challenges. The ultimate goal of disease detection is to quantify the occurrence level of disease by evaluating the incidence rate of disease (disease leaf/spike/plant ratio) and disease severity (damaged area). To assess the incidence rate of disease, we need to detect the damaged leaves accurately. Assessing the disease severity requires the accurate calculation of crop damage area. Therefore, accurate detection of diseases and a more compact indication of the scope of diseases are the premise of quantifying the occurrence level of diseases. However, in real-world applications, the orientation of the wheat stripe rust regions is arbitrary, and applying the horizontal bounding boxes (HBBs) to oriented object detection would lead to excessive redundant background regions in the HBB, as illustrated in Figure 1. This is not conducive to accurately calculating the damaged area of leaves to determine the severity of wheat stripe rust in future work. Additionally, since the arbitrary-oriented wheat stripe rust regions generally have a large aspect ratio, the use of general horizontal detectors tends to produce missing detection. This is not conducive to the accurate calculation of wheat stripe rust incidence rate in future work. Finally, multiple horizontal boxes overlap, making it difficult for farmers or agricultural experts to examine the real location of the disease. So, the horizontal detection methods cannot be directly applied in wheat stripe rust detection.

To solve the abovementioned problems, the idea of arbitrary-oriented object detection proposed in the fields of text detection (Zhou et al., 2017; Liao et al., 2018; Yuan et al., 2020) and remote sensing image detection (Yang et al., 2019b; Zhang et al., 2019; Yi et al., 2020) was used for reference, i.e., adding angle prediction on the basis of general object detector can realize the accurate positioning of object instances in any direction. This method can further refine the horizontal circumscribed rectangle of object predicted by the general object detector, and calculate the minimum area of the object circumscribed rectangle, which is conducive to estimating the damaged area of the disease in future work. At the same time, it can improve the performance of the algorithm for dense disease detection in complex scenes. We developed WSRD-Net, a refined single-stage oriented detector based on RetinaNet (Lin et al., 2017). On the basis of RetinaNet, it adds the feature refinement module (FRM) and designs the horizontal-to-oriented anchor mechanism. However, most of the existing disease images in public datasets are based on horizontal annotation, which is unsuitable for our WSRD-Net method. To advance the research of wheat stripe rust detection, we established the WSRD2021, a new in-field wheat stripe rust dataset based on oriented annotation in this work. We conducted extensive experiments on the WSRD2021 dataset, and the detection results of our WSRD-Net method outperform other state-of-the-art detection frameworks on the WSRD2021 dataset. The main contributions of this work include the following three aspects:

(1) WSRD-Net is developed to detect wheat stripe rust, which can realize precise localization of arbitrary-oriented wheat stripe rust.
(2) WSRD-Net can solve the feature misalignment problem for wheat stripe rust with a large aspect ratio detection by introducing FRM and using horizontal-to-oriented anchor mechanism to improve speed and accuracy.
(3) A new in-field wheat stripe rust dataset WSRD2021 based on oriented annotation is established to demonstrate the effectiveness of the developed method as well as build a benchmark for subsequent works.

## Materials and Methods

### Wheat Stripe Rust Dataset Construction

**Figure 2:**
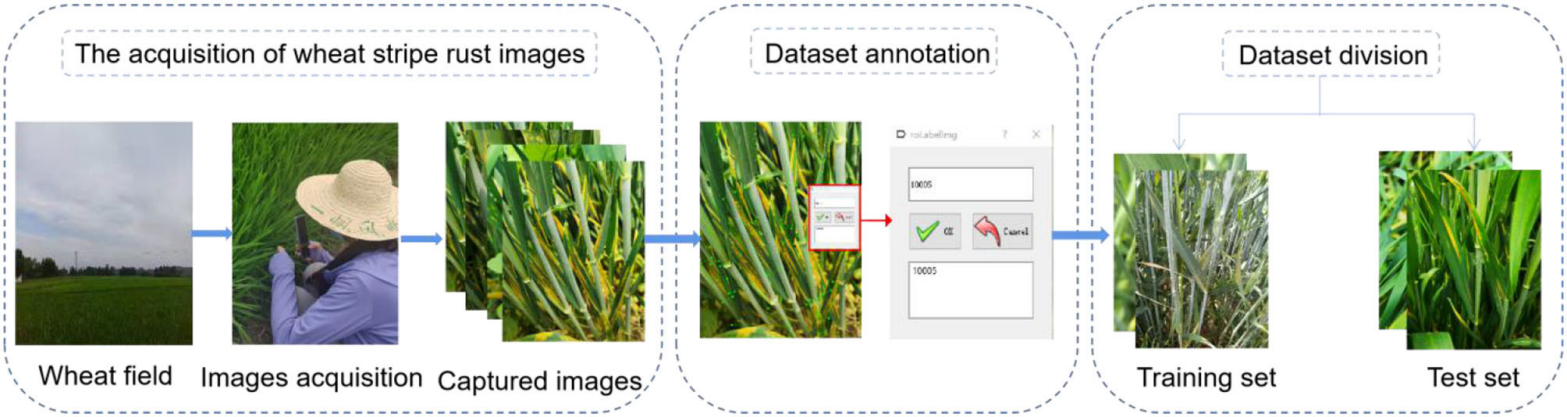
Flowchart of dataset establishment.

The dataset construction includes the acquisition of wheat stripe rust images, dataset annotation, and dataset division. A flowchart of dataset establishment is shown in Figure 2.

#### In-field Wheat Stripe Rust Images Acquisition

Wheat stripe rust images were collected in wheat fields in various counties in the Anhui Province. Images of wheat stripe rust were collected around the middle of April every year, as the disease was in the incidence stage at that time. Some human and material resources were devoted to collecting wheat stripe rust images. RGB images of wheat stripe rust were taken from the front and back of wheat leaves using a mobile phone because the symptoms of wheat stripe rust on the front and back of the leaves are different. Disease images were saved in a JPG format, and we selected 979 high-quality images to establish our dataset.

#### Oriented Object Annotation of Wheat Stripe Rust Images

**Figure 3:**
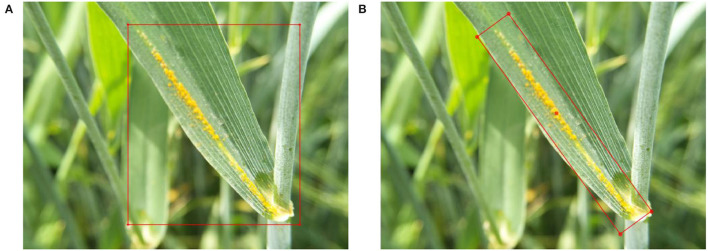
Object annotation. **(A)** Horizontal object annotation (HBB) and **(B)** oriented object annotation (OBB).

In general horizontal detectors, objects are always represented by HBB, as shown in Figure 3A. A HBB is usually represented by (*x, y, w, h*), where (*x, y*) represents the coordinates of the center point of the bounding box, *w* represents the width of the bounding box, and *h* represents the height of the bounding box. In most cases, HBB can accurately represent the location information of the object. In the above scenes, the objects can appear in various orientations and park densely. Therefore, an oriented bounding box (OBB) is introduced to accurately represent objects with arbitrary directions in dense scenes. As shown in Figure 3B, an object is defined as OBBs with five parameters (*x, y, w, h*, θ). The coordinate (*x, y, w, h*) represents the geometric center, height, and width of the bounding box. The θ represents the angle of the long side to the x-axis. Data labeling is performed by a professional plant protection personnel using roLabelImg software (Cgvict, 2017). The labeled disease location coordinates and category are saved as an XML file.

**Figure 4:**
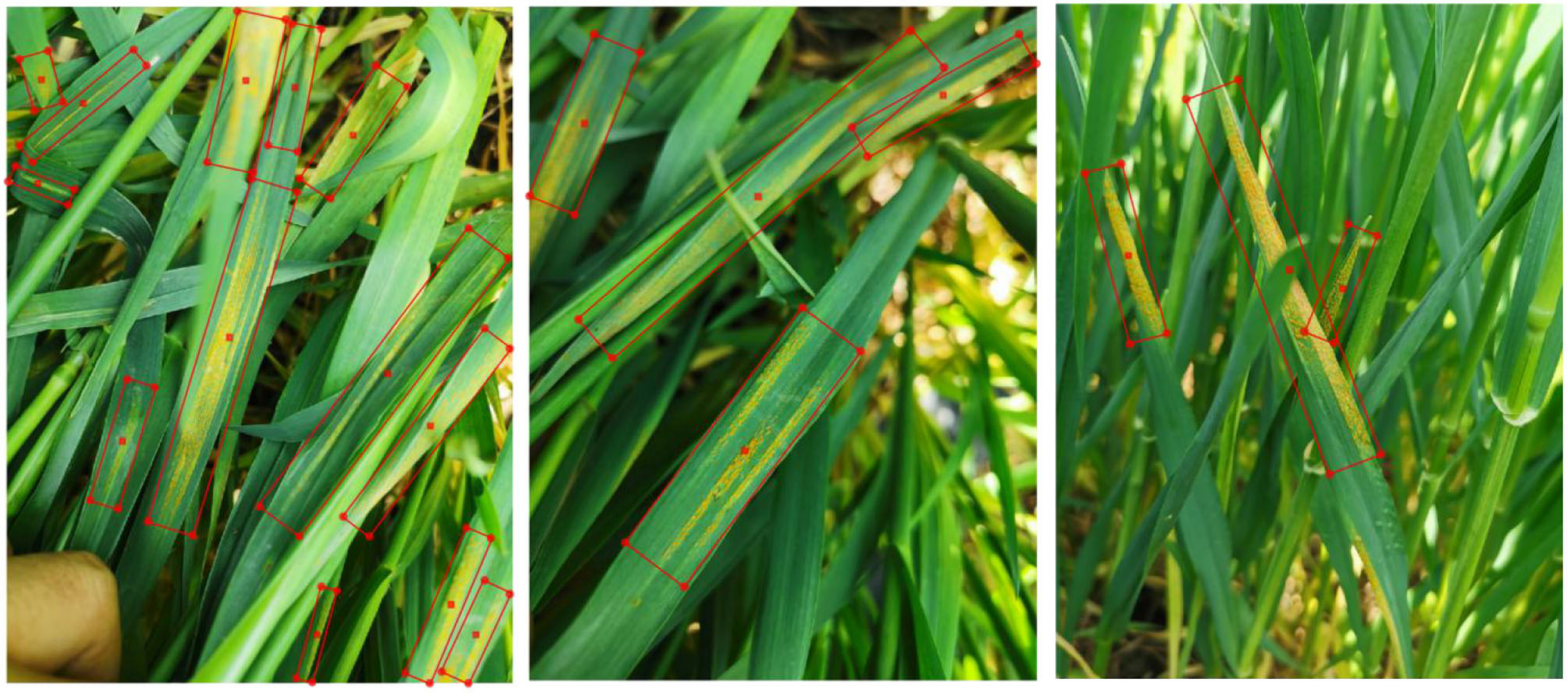
Some examples of WSRD2021 dataset.

After image annotation, the collected dataset is randomly divided into training and testing sets with the ratio to the total images of 0.8 and 0.2, respectively. Then, the dataset of collected images is named as Wheat Stripe Rust Dataset 2021 (WSRD2021), and some examples of WSRD2021 are illustrated in Figure 4.

#### Characteristics of Data

**Figure 5:**
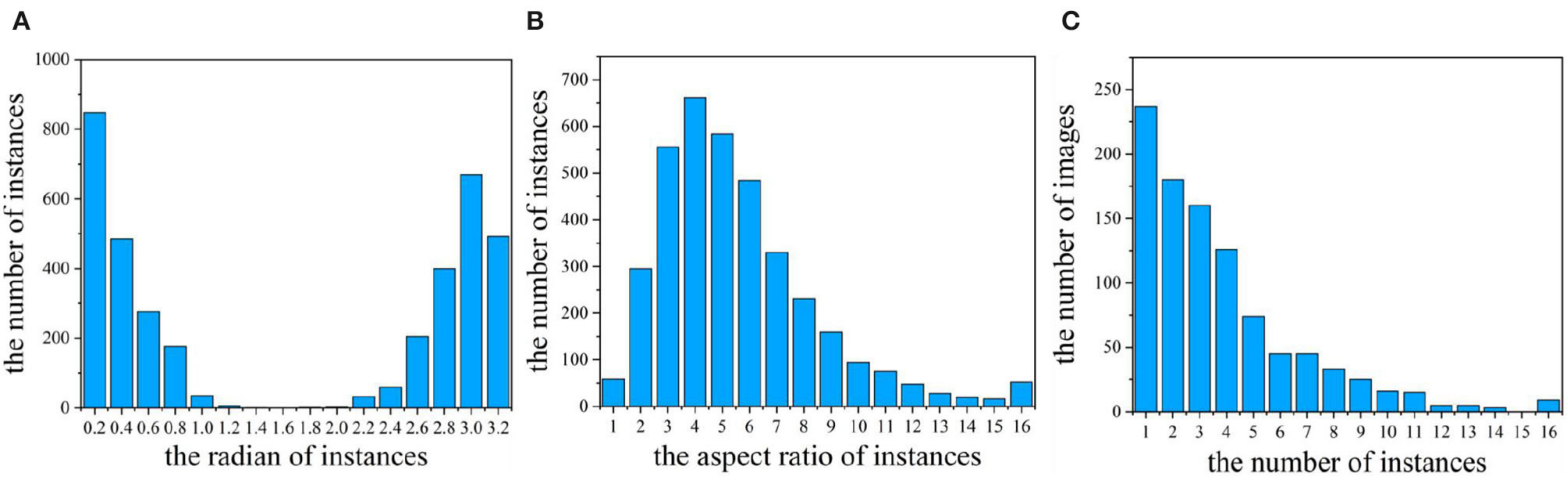
Characteristics of dataset. **(A)** Orientation histogram for all instances; **(B)** aspect ratio histogram for all instances; and **(C)** number of instances for all images.

Wheat stripe rusts on wheat leaves have a high diversity of orientations. As shown in Figure 5A, the regions of wheat stripe rust have arbitrary angles in (0°, 180°). The arbitrary angle distribution of WSRD2021 provides a new dataset for oriented object detection research.

In oriented object detection, the aspect ratio of the instance greatly influences the detection result. We calculate the aspect ratios of each instance in our dataset, as shown in Figure 5B. The stripe rust spots of wheat in our dataset are slender, and the aspect ratios are large. We can observe that the aspect ratio of instances in our dataset varies greatly. Moreover, more than 75% of the instances have a large aspect ratio (aspect ratio >3) in our dataset.

The number of instances per image is an important property for the object detection dataset. In our dataset, the number of instances in the image is related to the severity of the occurrence of wheat stripe rust. The more severe the wheat stripe rust is in the wheat field, the more instances are present in the image. We count the number of instances in each picture, and the result is shown in Figure 5C. It can be seen that the number of instances per image in our dataset varies widely, and the instance can be very dense (up to 16 instances per image) or very sparse (only one instance per image). Moreover, more than 60% of the images have multiple instances (the number of instances ≥ 3).

### Proposed Method

**Figure 6:**
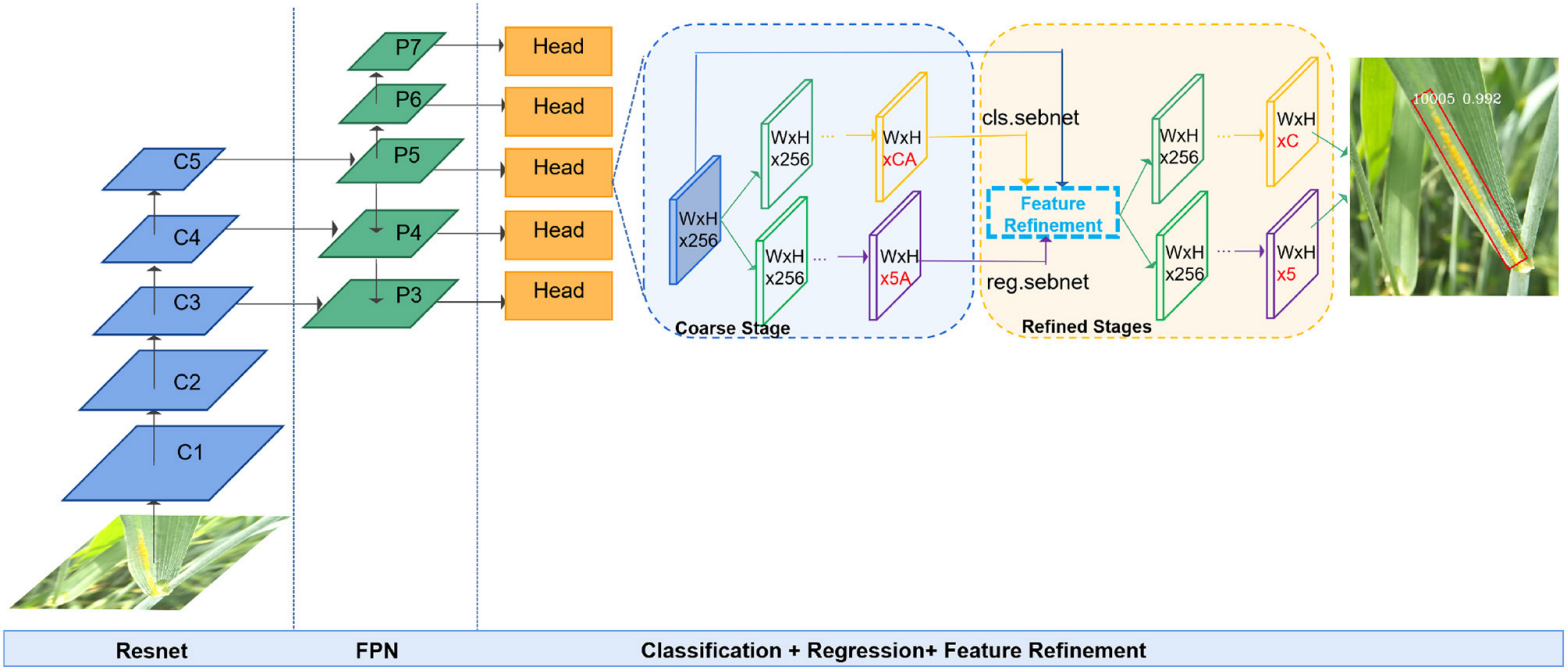
Schematic representation of WSRD-Net framework. The model uses ResNet as a backbone and builds a feature pyramid network (FPN) on top of the ResNet architecture. Two task-specific subnetworks and FRM. “A” denotes the number of anchors on each feature point, “C” denotes the number of categories, and “5” indicates the coordinates and orientation of the bounding box.

R^3^det (Yang et al., 2019a) model, whose FRM can solve the feature misalignment problem, is very suitable for detecting wheat stripe rust with large aspect ratios, dense distribution, and arbitrary orientations. Therefore, the R^3^det model is used for wheat stripe rust detection. Figure 6 shows a schematic representation of WSRD-Net framework used in this study. WSRD-Net is a refined, single-stage oriented detector based on the RetinaNet (Lin et al., 2017), consisting of a single FCN comprised of a ResNet-FPN backbone network, two task-specific subnetworks, and the FRM. The backbone is responsible for extracting a multiscale feature map over the original image *via* a set of basic conv + relu + pooling layers. The first subnet is used to perform object classification, and the second subnet performs bounding box regression. The FRM is used to continuously refine the predicted bounding box to improve the regression accuracy. The whole process includes two stages, namely, the coarse stage and the refined stage. In the coarse stage, the horizontal anchors are used to regress oriented anchors, and then, the oriented anchors are used in the subsequent refined stages to obtain the final detection results under arbitrary orientations.

#### Horizontal-to-Oriented Anchor Mechanism

At present, most general detectors use the horizontal anchor. The horizontal anchor based approach has the advantage of using the horizontal circumscribing rectangle of the ground-truth to calculate intersection-over-union (IoU), which can reduce the use of anchor points and match more positive samples, but introduces a large number of non-object or regions of other objects. Many efficient oriented object detection methods use the oriented anchor to better locate the object in any direction and avoid introducing non-object areas. The setting of horizontal anchors uses scale and aspect ratio parameters. For the detector based on oriented anchor, more studies add several rotation angles for each anchor on the basis of scale and aspect ratio parameters to obtain oriented anchors. However, this setting increases the number of anchors, thereby reducing the efficiency of the model.

**Figure 7:**
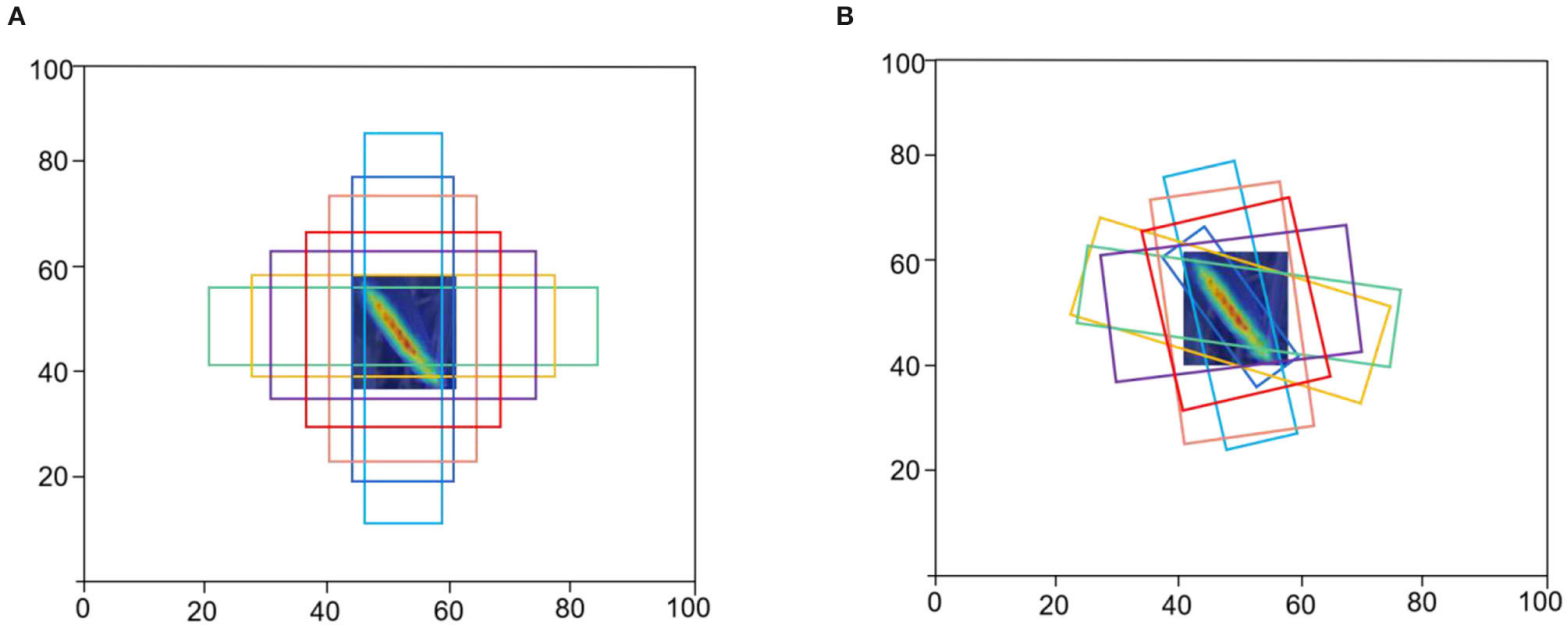
Example of horizontal anchors and refined oriented anchors. **(A)** Horizontal anchors of the coarse stage and **(B)** oriented anchors are used in the refined stage.

Considering the advantages and disadvantages of horizontal and oriented anchors, WSRD-Net adopts horizontal-to-oriented anchor mechanism. In the coarse stage, horizontal anchors (Figure 7A) are first generated from predefined anchors to improve the speed and generate more candidate boxes. Assume that one anchor is denoted as a 5-tuple (*x, y, w, h*, θ_0_), where (*x, y*) indicates the geometric center. The width *w* is set to the horizontal side, and the height *h* is set to the vertical side, θ_0_ is 0. We obtain the oriented anchor by learning the offset of the five parameters of the horizontal anchor and the oriented ground-truth. In the subsequent refined stage, the oriented anchors (Figure 7B) are used for regression to obtain more accurate location.

In WSRD-Net, the size of the horizontal anchor changes from 32 × 32 to 512 × 512 on the P3-P7 characteristic diagram of the pyramid. At each pyramid level, the aspect ratio of the anchor is (1, 1/2, 2, 1/3, 3, 1/5, 5), and the scale is (2^0^, 2^1/3^, 2^2/3^).

#### Oriented Bounding box

To predict objects, the previous general detectors represent horizontal rectangle by four parameters (*x, y, w, h*) and regress horizontal rectangle. In this study, five parameters (*x, y, w, h*, θ) are used to represent an arbitrary-oriented rectangle. Therefore, it calls for predicting an additional angle offset in the regression subnet, and then, the learning target is calculated as:


(1) $$\begin{array}{l} \;t^{*}{\;}_{x}=(x^{*}-x_{a})/w_{a},t^{*}{\;}_{y}=(y^{*}-y_{a})/h_{a}\\ t^{*}{}_{w}=\log(w^{*}/w_{a}),t^{*}{\;}_{h}=\log(h^{*}/h_{a}),t^{*}{\;}_{\theta }=\theta^{*}-\theta_{a}\\ \;t_{x}=(x-x_{a})/w_{a},t_{y}=(y-y_{a})/h_{a}\\ \;t_{w}=\log(w/w_{a}),t_{h}=\log(h/h_{a}),t_{\theta }=\theta /\theta_{a} \end{array}$$

where *x, y, w, h*, θ denote the center coordinates of the box, width, height, and angle, respectively. Variables $x^{*},\;x_{a},\;x\;$represent values related to the ground-truth box, anchor box, and predicted box, respectively; the same is applicable for *y, w, h*, and θ.

#### Feature Refinement Module

In many proposed refined detectors, the same feature map is used to calculate multiple classifiers and regressors. However, the location changes of the bounding box will cause feature misalignment. Feature misalignment occurs during the refinement process of bounding box, resulting in inaccurate features, which can be disadvantageous for the detection of wheat stripe rust with a large aspect ratio.

**Figure 8:**
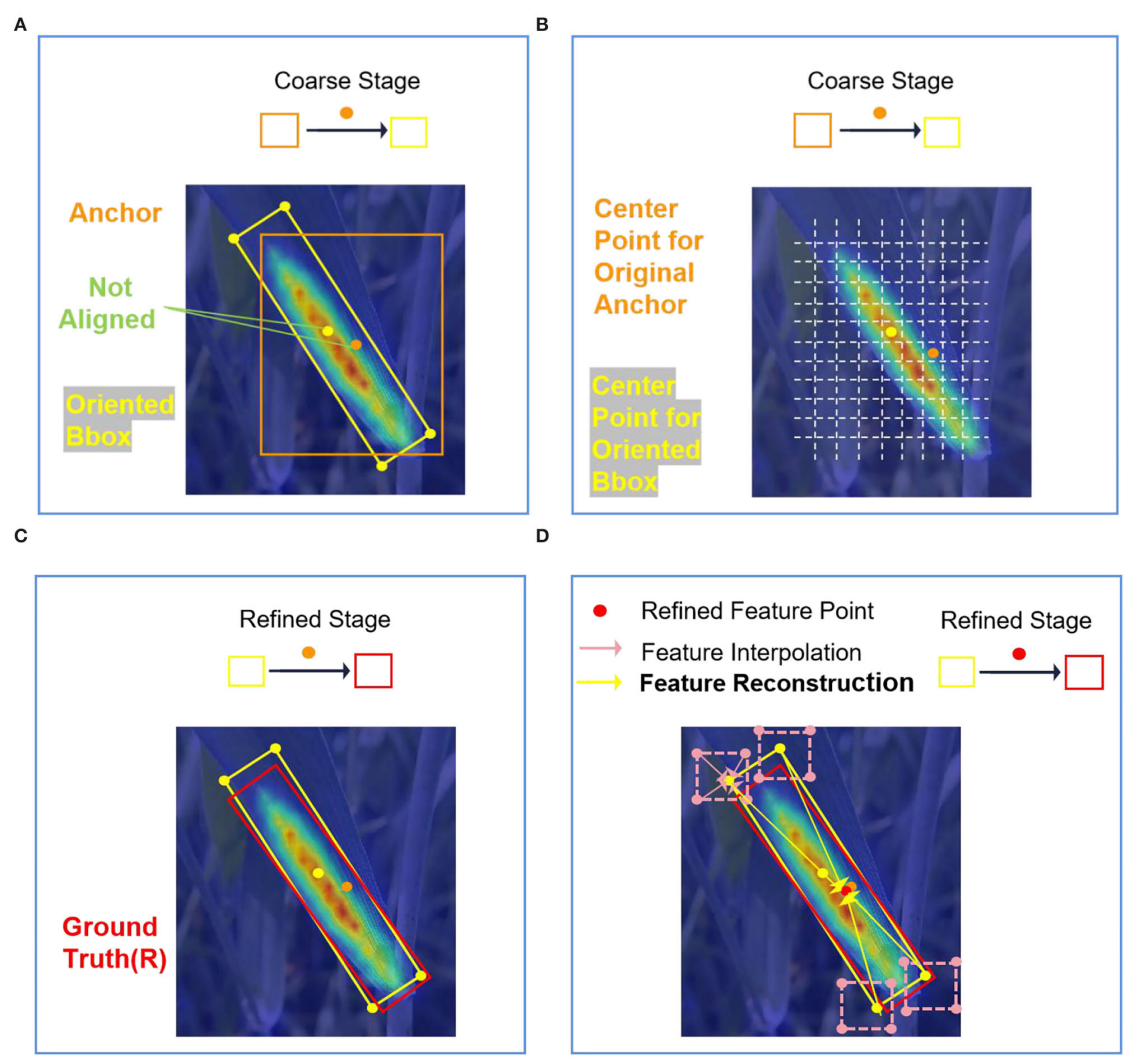
Feature misalignment and the core idea for FRM. **(A)** Feature misalignment of the coarse stage; **(B)** location of center points for original horizontal anchor and refined anchor in the feature grid; **(C)** not aligned feature is used in the refined stage; and **(D)** aligned features are used in the refinement stage by reconstructing the feature map.

Specifically, each feature point on the feature map corresponds to several anchors. One of the anchors can be considered for example, and the center of the anchor is the feature point on the feature map. As shown in Figure 8A, in the coarse stage, the original horizontal anchor (orange box) is regressed to the OBB (yellow one) as a result. From the feature grid (Figure 8B), we can see that the anchor feature is extracted from the orange point by using the full convolution network. However, the center point (yellow point) of the OBB is not aligned with the feature points (orange point) on the original feature map. If we use the original feature map in the refined stage, it means that the feature of the refined anchor is still extracted from the orange point, resulting in feature inconsistency (Figure 8C). The misalignment of anchor feature and anchor position will severely affect the detection performance. Therefore, the FRM is used to solve the problem of feature misalignment.

**Figure 9:**
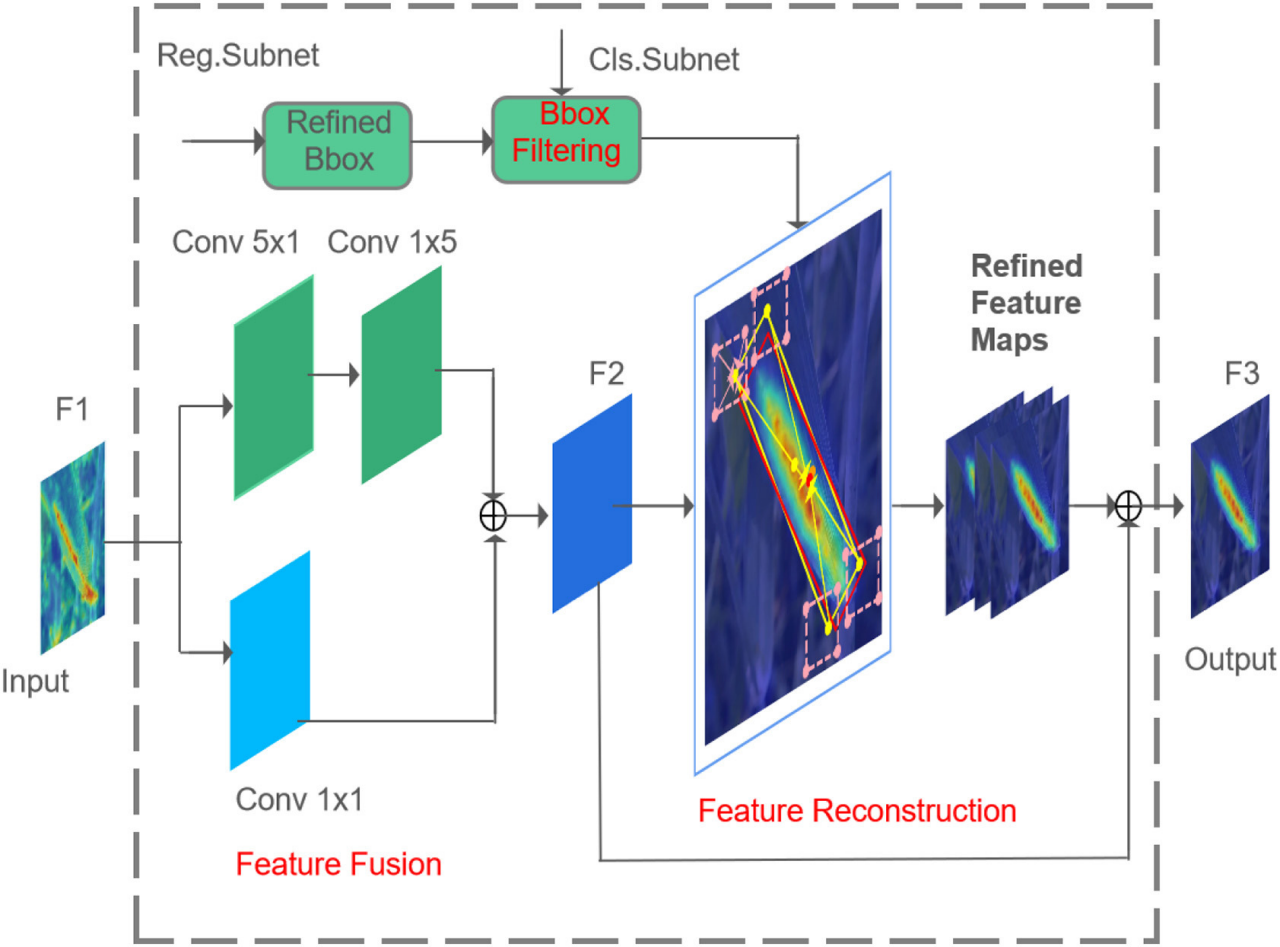
Feature refinement module.

The FRM can find the corresponding feature area of the current OBB by calculation, and achieve the purpose of feature alignment by feature map reconstruction, as shown in Figure 8D. The structure of the FRM is shown in Figure 9. The FRM consists of a feature fusion module and a feature reconstruction module. In the feature fusion module, the feature map F1 output from FPN is sent to two parallel convolution layers. The first one passes through a 5 × 1 convolutional layer and then passes through a 1 × 5 convolutional layer, and the second one is with one 1 × 1 convolutional layer. The 5 × 1 convolutional layer can better capture vertical features, which is good for detecting instances with height greater than width, while 1 × 5 convolutional layer can better capture horizontal features, which is suitable for detecting instances with width greater than height. The outputs of two feature maps from the two parallel convolution layers are fused into a new feature map F2 by element-wise addition.

**Figure 10:**
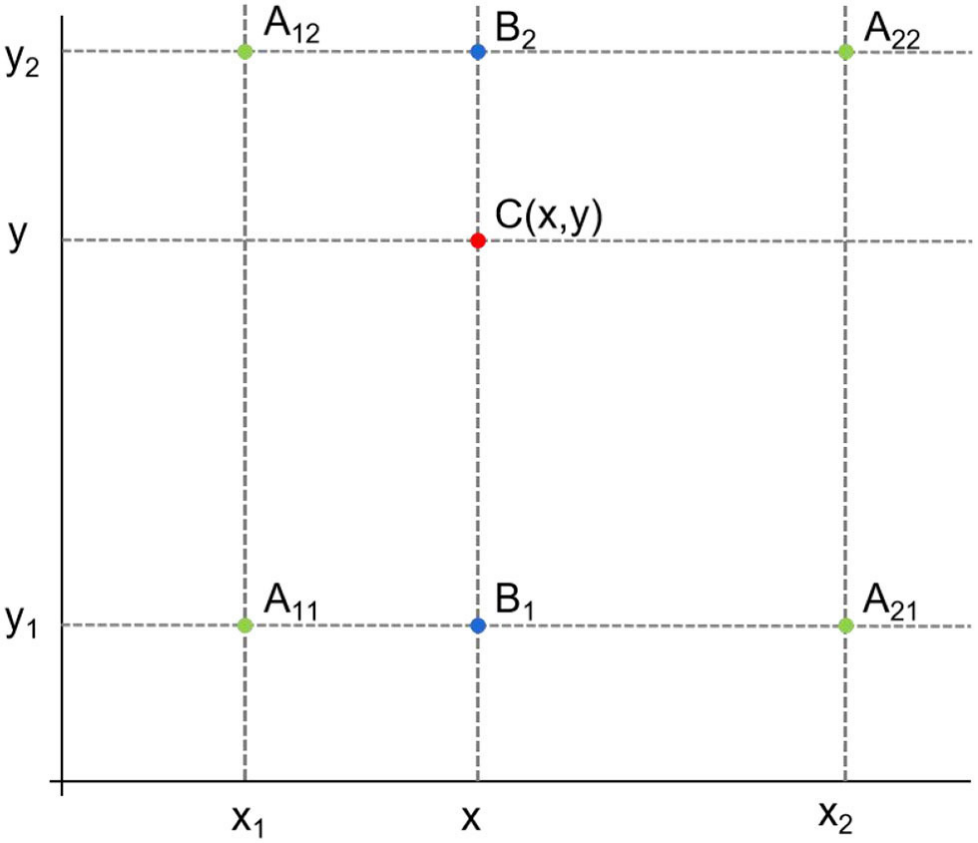
Bilinear feature interpolation.

Some redundant OBBs generated from the coarse stage are discarded by bounding box filtering, that is, only the OBB with the highest score of each feature point is retained. The retained OBB and feature map F2 act as input in the feature reconstruction module, and the reconstructed feature map F3 acts as output. Specifically, the retained OBBs (one center point and four corner points) are mapped back to the feature map F2 by bilinear interpolation, and the feature vector of the corresponding feature point is obtained. Then, each feature vector is superimposed on the original feature map F1 for fusion to obtain the refined feature map. Finally, the refined feature map and feature map F2 are fused to obtain the reconstructed feature map F3. To accurately obtain the location feature information of the OBB, the bilinear feature interpolation method is adopted, as shown in Figure 10. According to the known values of *A*_11_ = (*x*_1_, *y*_1_), *A*_12_ = (*x*_1_, *y*_2_),_*A*_21_ = (*x*_2_, *y*_1_)_ and *A*_22_ = (*x*_2_, *y*_2_), the point *C* (*x, y*) to be interpolated is calculated. First, *B*_1_ and *B*_2_ are obtained by linear interpolation in the X direction, and then *C* (*x, y*) is obtained by linear interpolation in the Y direction. Feature interpolation can be formulated as follows:


(2) $$\begin{array}{l} f\left(x,y\right)=\frac{f\left(A_{11}\right)}{\left(x_{2}-x_{1}\right)\left(y_{2}-y_{1}\right)}\left(x_{2}-x\right)\left(y_{2}-y\right)\\ \;+\frac{f\left(A_{21}\right)}{\left(x_{2}-x_{1}\right)\left(y_{2}-y_{1}\right)}\left(x-x_{1}\right)\left(y_{2}-y\right)\\ \;+\frac{f(A_{12})}{\left(x_{2}-x_{1})(y_{2}-y_{1}\right)}(x_{2}-x)(y-y_{1})\\ \;+\frac{f(A_{22})}{\left(x_{2}-x_{1})(y_{2}-y_{1}\right)}(x-x_{1})(y-y_{1}) \end{array}$$

where *f*(*A*_11_) represents the feature vector of the point *A*_11_, the same as *f*(*A*_12_), *f*(*A*_21_), and *f*(*A*_22_). *x*_1_, *y*_1_ denote the coordinate value of point *A*_11_ on the X and Y axes, *x*_1_, *y*_2_ denote the coordinate value of point *A*_12_ on the X and Y axes, *x*_2_, *y*_1_ denote the coordinate value of point *A*_21_ on the X and Y axes, and *x*_2_, *y*_2_ denote the coordinate value of point *A*_22_ on the X and Y axes.

#### Skew IoU

**Figure 11:**
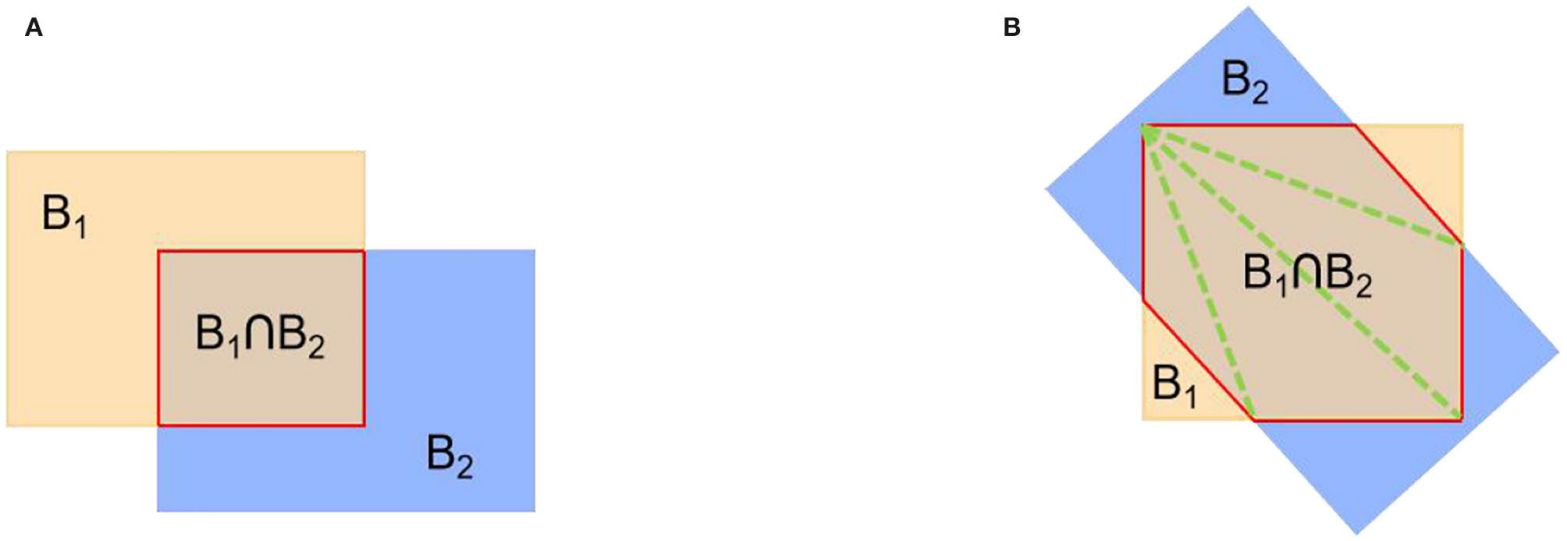
Examples of standard IoU and S-IoU computation. **(A)** Standard IoU and **(B)** S-IoU.

General horizontal object detection methods use IoU computation for the axis-aligned strategy to choose the positive samples. The overlap image of the two HBBs is a rectangle, as shown in Figure 11A. However, the OBB in oriented object detection can be generated in any orientation. The overlap of the two OBBs may be irregular polygons, as shown in Figure 11B. Thus, using the IoU computation method like in general horizontal object detection may lead to an inaccurate IoU of the skew interactive bounding box, and further ruin the bounding box prediction. Therefore, we use skew IoU (S-IoU) (Ma et al., 2018) to solve this problem; the overlapping area (*B*_1_∩*B*_2_) of two oriented rectangles is calculated by triangulation method. The S-IoU between two oriented rectangles can be calculated by the following equation:


(3) $$\begin{array}{l} \text{S-IOU}=\frac{area\left(B_{1}\cap B_{2}\right)}{area\left(B_{1}\cup B_{2}\right)} \end{array}$$

where *B*_1_ and *B*_2_ represent two oriented rectangles.

#### Loss Function

**Figure 12:**
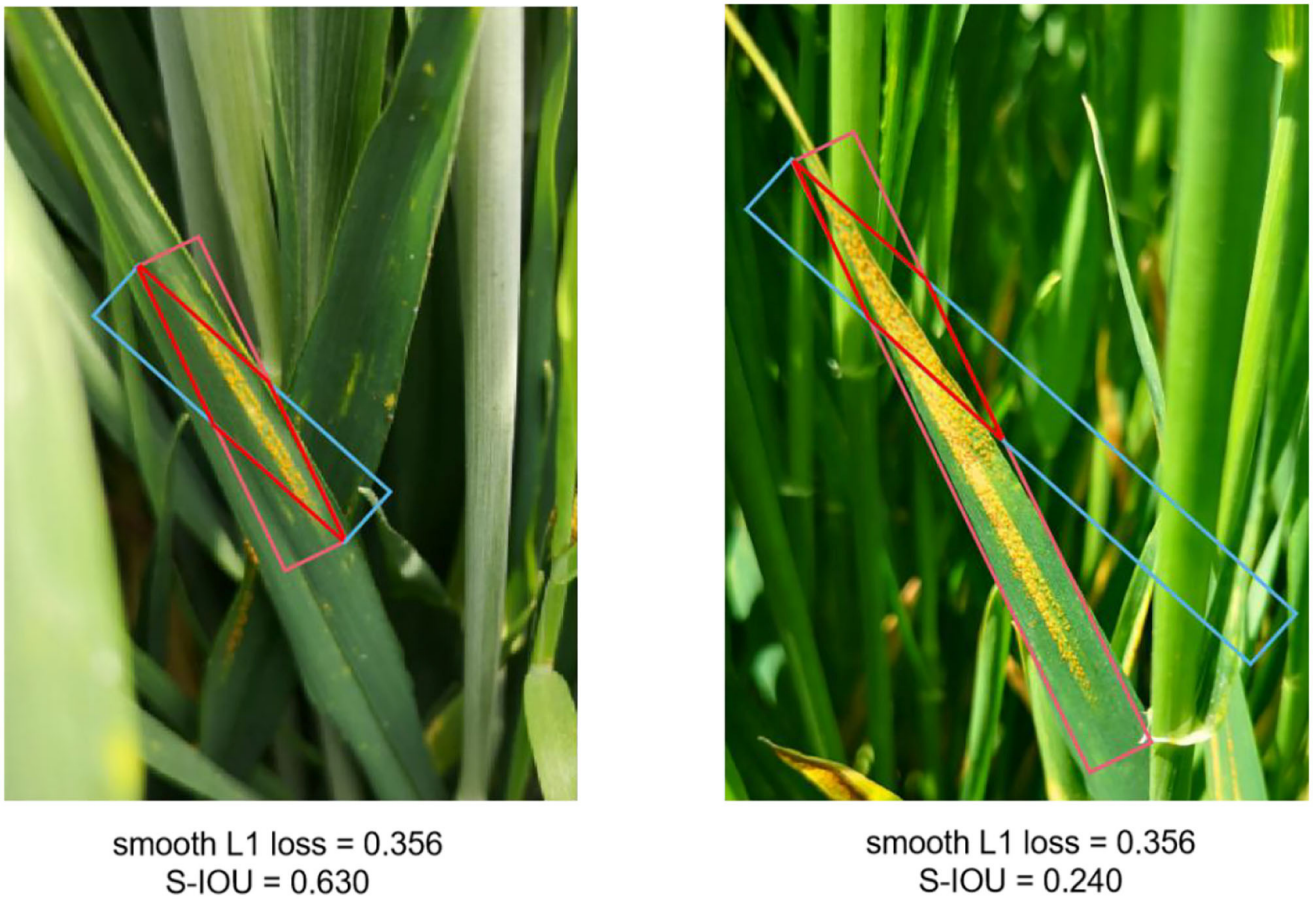
Comparison between S-IoU and Smooth L1 loss.

In our wheat stripe rust dataset, there are many objects with large aspect ratios. These wheat stripe rusts with a large aspect ratio are sensitive to S-IoU. As shown in Figure 12, the predicted OBB and oriented ground-truth of wheat stripe rust in each group have the same height and width. The two groups have the same change in angle and different aspect ratios. As a result, the two groups have the same smooth L1 loss value and a quite different S-IoU.

To train WSRD-Net, we adopted a new derivable approximate S-IoU loss function. Compared with the standard smooth L1 loss function, the new loss can obtain more accurate rotation estimation. In particular, the angle loss is added to the regression loss, and the multi-task loss function of WSRD-Net is defined as follows:


(4) $$\begin{array}{l} L=\frac{\lambda_{1}}{N}\underset{i}{\sum }o_{i}\frac{L_{reg}\left(v_{i},{v^{*}}_{i}\right)}{\left|L_{reg}\left(v_{i},{v^{*}}_{i}\right)\right|}|f\left(\text{S-IOU}\right)|+\frac{\lambda_{2}}{N}\underset{i}{\sum }L_{cls}\left(p_{i},{p^{*}}_{i}\right) \end{array}$$

(5) $$\begin{array}{l} L_{reg}\left(v,v^{*}\right)=L_{smooth-l1}\left(v_{\theta },{v^{*}}_{\theta }\right)-IOU\left(v_{\left\{x,y,w,h\right\}},{v^{*}}_{\left\{x,y,w,h\right\}}\right) \end{array}$$

where *i* is the index of anchor, *N* is the number of anchors, and the value of *o*_*i*_ represents the binary value (*o*_*i*_ = 1 is foreground and *o*_*i*_= 0 is background). *v* is the predicted offset vector, and v^*^ is the target vector of ground-truth. The value of ${p^{*}}_{i}\;$represents the ground-truth label, *p*_*i*_ is the classification confidence of i-th sample. (*x, y, w, h*, θ) represent the center coordinates of the box, width, height, and angle, respectively. *S-IOU* is the overlap of the predicted OBB and oriented ground-truth. The default values are λ_1_ =1 and λ_2_ =1. The classification loss *L*_*cls*_ is focal loss (Lin et al., 2017). The function *f*(*S-IOU*) = 1−*S-IOU* is the loss function related to S-IoU. The function *IOU*(.) is a HBB IoU calculation function.

#### Evaluation Metrics

The S-IoU between oriented boxes is used to distinguish detection results. If the S-IoU between a detection bounding box and a ground-truth is higher than a given threshold, then the detection box is considered to be true-positive (TP), otherwise, it is considered to be false-positive (FP). If a ground-truth box has no matching detections, it is considered to be a false-negative (FN). The Precision and Recall are then computed by the following equations:


(6) $$\begin{array}{l} P=\frac{TP}{FP+TP} \end{array}$$

(7) $$\begin{array}{l} R=\frac{TP}{FN+TP} \end{array}$$

where *P* is Precision; *R* is Recall; *TP* is the total number of correctly detected wheat stripe rusts; *FP*+*TP* represents the total number of detected wheat stripe rusts; *FN*+*TP* represents the total number of true wheat stripe rusts.

The Precision-Recall (P-R) curve, widely known as the P-R curve for evaluating the performance of an object detection algorithm, was used to assess the models. The area under this precision-recall curve is the Average Precision (AP). In this study, AP is selected as the primary evaluation metric in comparison experiments. We also apply the other two metrics to evaluate the performance of all detection models: Recall and FPS.

## Experimental Results

### Implementation Details

Pytorch framework over python 3.7 was used to implement the developed network architectures of WSRD-Net and all other state-of-the-art models. Models were trained on an Ubuntu 18.04 server with one 24GB memory NVIDIA TITAN RTX GPU card. WSRD-Net and all other state-of-the-art models are trained for 24 epochs with a batch size of 4 examples. The initial learning rate for WSRD-Net is 4e-3, and the learning rate is reduced at the 8th, 16th, and 20th epoch. The initial learning rate of all other state-of-the-art models is 0.01, and the learning rate is reduced at the 12th, 16th, and 22nd epoch. Multiscale (1,333, 800), (1,700, 1,000), (2,000, 1,200) training is adopted in all models. The other parameters of models used in this study are consistent with their default parameters without any adjustment.

### Comparison Results

To verify the performance of WSRD-Net, the comparison experiments with the four state-of-the-art oriented object detection models, namely, Faster R-CNN OBB (Ding et al., 2021), RetinaNet OBB (Ding et al., 2021), Faster R-CNN OBB + Dpool (Dpool by replacing RoI Align in Faster R-CNN OBB) (Ding et al., 2021), and Faster R-CNN OBB + RT (Faster R-CNN OBB + RoI Transformer, RoI Transformer by replacing RoI Align in Faster R-CNN OBB) (Ding et al., 2021) are conducted. These models use ResNet50 as the backbone. The same dataset was used for training and testing the four models, respectively. Detection results are presented in Table 1; the AP of RetinaNet OBB, Faster R-CNN OBB, Faster R-CNN OBB + Dpool, and Faster R-CNN OBB + RT are 38.7, 56.7, 56.1, and 60.6%, respectively. The WSRD-Net has a significantly higher AP (60.8%) than the other four models, demonstrating that WSRD-Net performs better in detecting the wheat stripe rust. Additionally, we also evaluate the Recalls of WSRD-Net and the four state-of-the-art oriented object detection models. The Recall value (73.8%) of WSRD-Net is high for wheat stripe rust as summarized in Table 1.

**Table 1:** Detection results of WSRD-Net compared with the state-of-the-art oriented object detection models.

**Model**	**AP (%)**	**Recall (%)**
RetinaNet OBB	38.7	55.7
Faster R-CNN OBB	56.7	71.1
Faster R-CNN OBB + Dpool	56.1	71.1
Faster R-CNN OBB + RT	60.6	72.9
WSRD-Net	60.8	73.8

**Figure 13:**
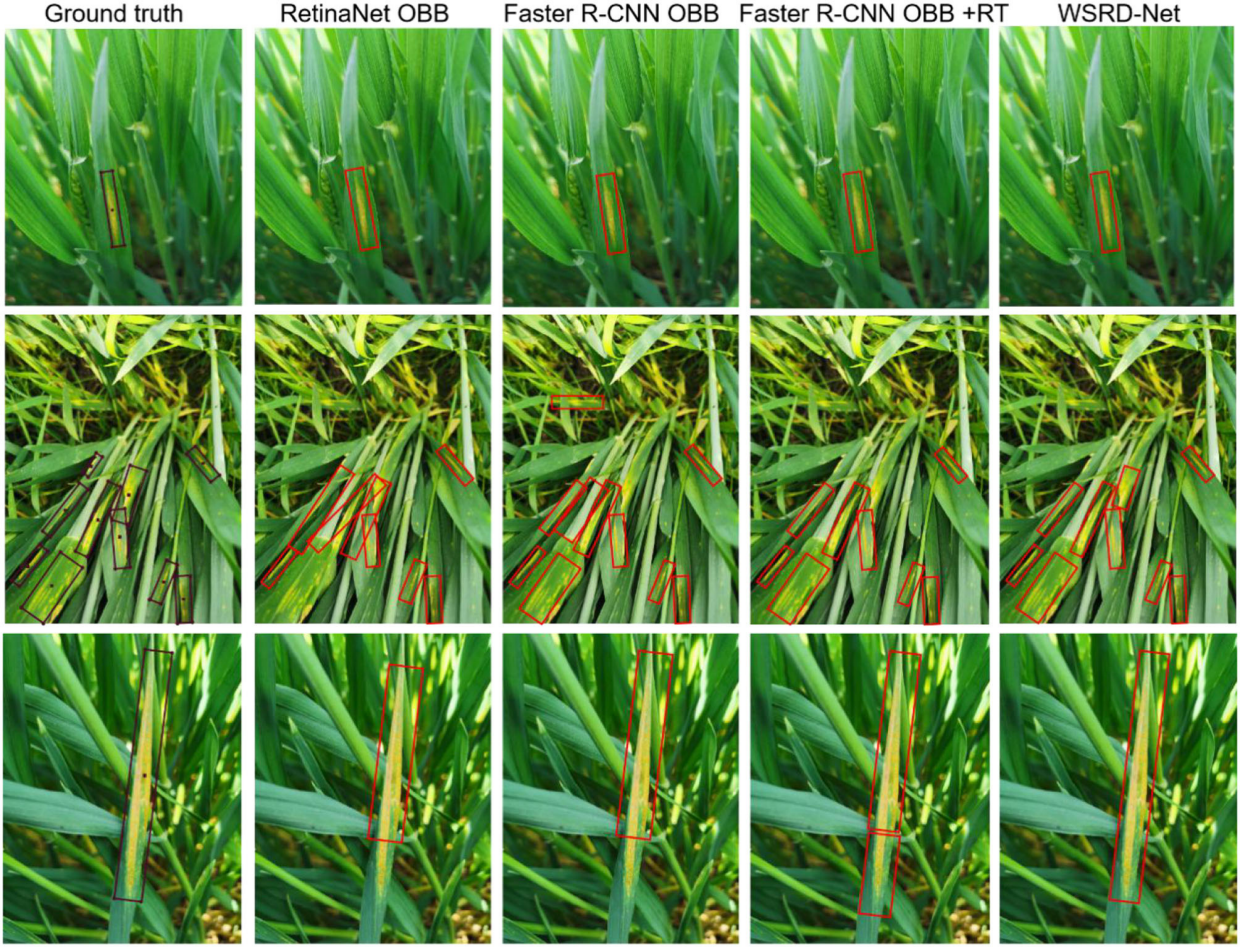
Visualization of the comparison results with other oriented object detection models.

Meanwhile, Figure 13 presents some examples of localization for wheat stripe rust areas by four different models based on oriented object detection. The ground-truth image in the first row only contains one diseased leaf, the ground-truth image in the second row contains multiple diseased leaves, and ground-truth image in the third row contains a leaf with a large aspect ratio. These examples show that the four oriented object detection models can effectively identify and locate the wheat stripe rust areas in natural fields. However, there are still some subtle differences. In the first row, all models perform well on the image containing only one wheat stripe rust. The second row of wheat stripe rust is densely distributed; WSRD-Net, Faster R-CNN OBB, and Faster R-CNN OBB + RT provide good detection results, while RetinaNet OBB has poor performance due to feature misalignment. In the third row, RetinaNet OBB and Faster R-CNN OBB result in a most partial localization for a large aspect ratio in the wheat stripe rust area. Faster R-CNN OBB + RT has two prediction bounding boxes on a single wheat stripe rust area. Only the WSRD-Net can accurately detect stripe rust with a large aspect ratio. Comparatively, WSRD-Net can more precisely locate the disease area and improve the overall performance due to the addition of FRM to solve the problem of feature misalignment.

### Comparison With the Other Backbones

To further verify the effectiveness of WSRD-Net approach, four influential backbones, including ResNet50, ResNet101 (He et al., 2016), ResNext50, and ResNext101 (Xie et al., 2017), were used for comparative experiments. The AP and Recall under four backbones are summarized in Table 2. It can be observed that WSRD-Net using ResNet50 and ResNet101 as backbone achieves the best performance with the highest AP (60.8%). WSRD-Net using ResNet101 as backbone achieves the highest Recall (75.3%). Among these results, the lowest AP occurs in using ResNext101 as the backbone. Network deepening does not significantly improve the performance of the model, mainly because the shallow convolution layer extracts relatively small features, and extensive information is extracted by deeper convolution. However, the wheat stripe rusts of our dataset are relatively small, there is no need to use high-level semantic information, and ResNet50 is enough.

**Table 2:** AP and Recall comparison with the other backbone.

**Backbone**	**AP (%)**	**Recall (%)**
ResNet50	60.8	73.8
ResNet101	60.8	75.3
ResNext50	51.9	69.2
ResNext101	51.6	69.0

By comparing the AP and Recall, it can be seen that using ResNet101 as the backbone performs best on our dataset. Considering the deep layers of ResNet101 network will reduce the detection efficiency, so we choose ResNet50 as the backbone of WSRD-Net.

### Comparison With Horizontal Detection Models

**Figure 14:**
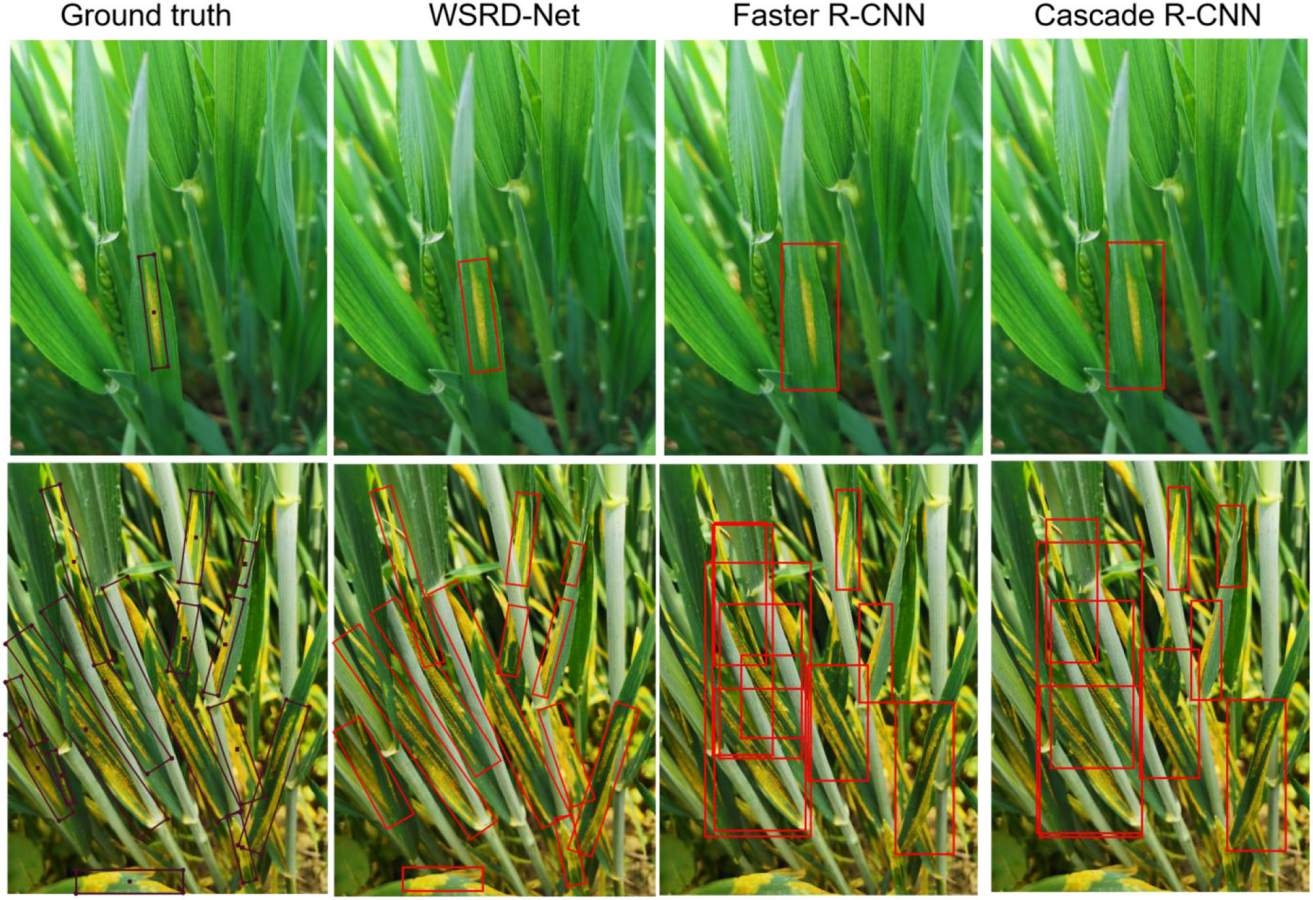
Visualization of the comparison results with horizontal detection models.

The visualization of detection results between the horizontal detection models and WSRD-Net is given in Figure 14. Although the horizontal detection model can effectively identify and locate the wheat stripe rust in the natural field, it also contains excessive redundant background area. As the image only contains one diseased leaf in the first row, the Faster R-CNN (Ren et al., 2017) and Cascade R-CNN (Cai and Vasconcelos, 2018) models accurately detect diseased areas. In the second row, as the image contains multiple diseased leaves, the large aspect ratio of wheat stripe rust, and park densely, the NMS algorithm using a HBB tends to produce missing detection. In addition, multiple horizontal boxes overlap, making it difficult to see the real location of the disease. Comparatively, WSRD-Net can more accurately locate the wheat stripe rust, especially in most images containing multiple diseased leaves. Experimental results show that the performance of WSRD-Net in detecting the wheat stripe rust is superior to the horizontal detection models.

### Detection Efficiency

We have compared the speed of WSRD-Net with the state-of-the-art oriented detection models and horizontal detection models on our dataset. All models adopt multi-scale (1,333, 800), (1,700, 1,000), (2,000, 1,200) training, and single-scale (1,333, 800) testing, and the result is summarized in Table 3. For comparison with the state-of-the-art oriented detection methods, when the same backbone (ResNet50) is applied, the Faster R-CNN OBB + RT and Faster R-CNN OBB + Dpool methods have a little lower speed, and WSRD-Net and RetinaNet OBB models achieve the relatively high speed (6.8 and 7.0 fps, respectively). The two-stage algorithm based on RoI operation does not share the amount of computation, which limits the computational efficiency of the two-stage algorithm, so WSRD-Net is faster than the two-stage algorithm. Furthermore, although WSRD-Net adds FRM module based on RetinaNet, the speed of WSRD-Net is only slightly lower than RetinaNet OBB. This is because the FRM module added in WSRD-Net filters out some redundant boxes by the bounding box filtering mechanism after the coarse stage, reducing the number of boxes, thus speeding up the model. Table 3 also summarizes the speed of oriented detection models and that of the horizontal detection models, namely, Faster-RCNN and Cascade R-CNN. Due to different R-anchor strategies and other network designs of oriented detection models, the network becomes more complex. We can observe that oriented detection models act twice more than the Faster-RCNN and Cascade R-CNN approaches.

**Table 3:** Speed comparison with the state-of-the-art oriented detection methods and horizontal detection models.

**Model no**.	**Speed (fps)**
WSRD-Net	6.8
RetinaNet OBB	7.0
Faster R-CNN OBB	6.7
Faster R-CNN OBB + Dpool	6.5
Faster R-CNN OBB + RT	6.6
Faster R-CNN	14.6
Cascade R-CNN	14.3

## Discussion

In this section, the comparison experiment between the WSRD-Net and state-of-the-art shows that the WSRD-Net is superior to the state-of-the-art (refer to Table 1 and Figure 13). In addition, the visualization of the detection result between the WSRD-Net and horizontal detection models indicates that the WSRD-Net is superior to the horizontal detection models (refer to Figure 14). WSRD-Net gets rid of the limitation of the non-maximum suppression (NMS) technique as post-processing, as it will result in missed detection of objects densely arranged in any direction along the horizontal line. This reduces the missing detection rate in dense scenes of wheat stripe rust, improves the detection accuracy, and helps to accurately calculate the incidence rate of wheat stripe rust. Compared with the horizontal detection method in previous studies, the orientation-based approach of WSRD-Net is able to obtain the minimum circumscribed rectangle of the damaged area with less background area, which is conducive to accurately counting the damaged area of leaves to determine the severity of wheat stripe rust in future work.

## Conclusion and Future Work

Accurate detection of wheat stripe rust is very important to improve wheat yield and quality. Due to the arbitrary-oriented wheat stripe rust and large aspect ratio, these issues bring big challenges for the task of detection of accurate wheat stripe rust. In this study, a wheat stripe rust detection method based on oriented object detection, WSRD-Net, is proposed for the detection of in-field wheat stripe rust. Also, we have built an oriented annotation dataset of in-field wheat stripe rust images, called the wheat stripe rust dataset 2021 (WSRD2021). We exploit five different oriented object detection models to perform wheat stripe rust detection on the newly collected in-field dataset WSRD2021. The optimal AP obtained by WSRD-Net is 60.8%, and the experimental results indicate that WSRD-Net can effectively detect the wheat stripe rust in the complex scenes, and is superior to the state-of-the-art. Moreover, the experimental comparison with horizontal detection models indicates that oriented object detection can lead to more accurate localization for disease areas than horizontal detection.

Despite the fact that we implement an oriented object detection method for the wheat stripe rust detection in the field and achieve successful performance in our dataset, there are some limitations for future study. First, our dataset only contains 979 images of a disease, with a single type of data and a small amount of data. So, our dataset needs to be extended to some different diseases of different crops sharing similar characteristics with current wheat stripe rust. For our wheat stripe rust dataset, the size difference of the disease area is large, and the edge characteristics are not obvious. So, WSRD-Net can be adapted or modified to better fit our wheat stripe rust dataset. Finally, WSRD-Net only pays attention to identifying disease categories and locating corresponding disease areas, but does not calculate the damaged area of the disease, which is also significant for monitoring of crop disease.

In the near future, we will concentrate on expanding the dataset and developing a more robust and powerful oriented object detection model based on the current model to fit our dataset. Moreover, we will design a method to calculate the damaged area of diseases to estimate the severity of crop diseases.

## Data Availability Statement

The datasets presented in this article are not readily available because the processed data required to reproduce these findings cannot be shared at this time as the data also forms part of an ongoing study. Requests to access the datasets should be directed to HL, liuhaiyun@mail.ustc.edu.cn.

## Author Contributions

HL: conceptualized experiment, selected algorithm, trained algorithms, collected and analyzed data, and wrote the manuscript. LJ, RW, and CX: were project supervisors. JD: analyzed the data. HC and RL: collected the data. All authors discussed and revised the manuscript. All authors contributed to the article and approved the submitted version.

## Funding

This work was supported in part by the National Natural Science Foundation of China (No. 32171888), the Natural Science Foundation of Anhui Higher Education Institutions of China (No. KJ2021A0025), the Major Science and Technology Projects in Anhui Province (No. 2020b06050001), and the National Key Research and Development Program of China (No. 2019YFE0125700).

## Conflict of Interest

The authors declare that the research was conducted in the absence of any commercial or financial relationships that could be construed as a potential conflict of interest.

## Publisher's Note

All claims expressed in this article are solely those of the authors and do not necessarily represent those of their affiliated organizations, or those of the publisher, the editors and the reviewers. Any product that may be evaluated in this article, or claim that may be made by its manufacturer, is not guaranteed or endorsed by the publisher.
